# Caryolan-1-ol, an antifungal volatile produced by *Streptomyces* spp., inhibits the endomembrane system of fungi

**DOI:** 10.1098/rsob.170075

**Published:** 2017-07-19

**Authors:** Gyeongjun Cho, Junheon Kim, Chung Gyoo Park, Corey Nislow, David M. Weller, Youn-Sig Kwak

**Affiliations:** 1 Division of Applied Life Science (BK21Plus), Institute of Agriculture and Life Science, Gyeongsang National University, Jinju 52828, Republic of Korea; 2 Forest Insect Pests and Diseases Division, National Institute of Forest Science, Seoul 02455, Republic of Korea; 3 Pharmaceutical Sciences, University of British Columbia, Vancouver, British Columbia, Canada; 4 US Department of Agriculture, Agricultural Research Service, Wheat Health, Genetics, and Quality Research, Pullman, WA, USA

**Keywords:** *Streptomyces*, volatile, antifungal activity, caryolan-1-ol, endomembrane system

## Abstract

*Streptomyces* spp. have the ability to produce a wide variety of secondary metabolites that interact with the environment. This study aimed to discover antifungal volatiles from the genus *Streptomyces* and to determine the mechanisms of inhibition. Volatiles identified from *Streptomyces* spp. included three major terpenes, geosmin, caryolan-1-ol and an unknown sesquiterpene. antiSMASH and KEGG predicted that the volatile terpene synthase gene clusters occur in the *Streptomyces* genome. Growth inhibition was observed when fungi were exposed to the volatiles. Biological activity of caryolan-1-ol has previously not been investigated. Fungal growth was inhibited in a dose-dependent manner by a mixture of the main volatiles, caryolan-1-ol and the unknown sesquiterpene, from *Streptomyces* sp. S4–7. Furthermore, synthesized caryolan-1-ol showed similar antifungal activity. Results of chemical-genomics profiling assays showed that caryolan-1-ol affected the endomembrane system by disrupting sphingolipid synthesis and normal vesicle trafficking in the fungi.

## 1. Introduction

The genus *Streptomyces* belongs to the phylum Actinobacteria and constitutes a group of soil-dwelling bacteria. *Streptomyces* exhibits morphological differentiation and is able to grow aerial hyphae and spore chains. Certain *Streptomyces* species and isolates are able to inhibit plant and animal pathogens from a wide range of terrestrial and aquatic environments [1]. This environmental flexibility has been attributed to the capacity of *Streptomyces* spp. to produce a wide diversity of secondary metabolites, which have been sources for new drugs with antiviral, antitumoral, antihypertensive, antibiotic and immunosuppressant activity [2–4]. These metabolites have been found in approximately 500 different groups of compounds from thousands of different strains of *Streptomyces.* About 10 400 of the known 33 500 bioactive microbial metabolites produced by microorganisms have been isolated from *Streptomyces* [5] and more than 60% of all antibiotics discovered have come from *Streptomyces* [6]. The genus *Streptomyces* is the largest source of bioactive metabolites, but the proportion of newly discovered metabolites from *Streptomyces* is decreasing [5]. For this reason, volatiles produced by *Streptomyces* have recently attracted much attention for their biomedical potential.

Volatiles are easily converted from liquids or solids into gas as a consequence of their low molecular weight (less than 300 Da) and high vapour pressure (0.01 kPa at 20°C). Because these properties allow them to diffuse easily, many bacterial volatiles function in competition, signalling and cooperation, but production is subject to environmental conditions. Bacterial volatiles belong to distinct compound classes, e.g. alkenes, alcohols, ketones, terpenes, benzenoids, pyrazines, acids and esters [7]. The biosynthesis of volatiles is generally linked to catabolic pathways including glycolysis, proteolysis and lipolysis [8,9]. Some volatiles also influence differentiation, growth, movement and stress response of plants, animals and fungi. For this reason, bacterial volatiles are of considerable research interest for a variety of applications. The volatiles dimethyl disulfide and dimethyl trisulfide have been reported to have powerful antifungal activity [10,11], however their exact mode(s) of action have not been determined at the molecular level [12–15].

In 1996, the *Saccharomyces cerevisiae* genome project was completed and a deletion mutant collection was developed. The development of molecular genetics has made the functional effect of chemicals in genetics easier to study using *S. cerevisiae*. Homozygous profiling (HOP) uses a pool of approximately 4700 diploid strains that deletes both copies of non-essential genes and predicts target pathway information of chemicals [16]. The pathways of drugs such as wortmannin, benomyl, tunicamycin, rapamycin, sulfometuron methyl, fluconazole, cycloheximide, FK506, caffeine, hydroxyurea [17], methyl methanesulfonate [18] and their potential targets have been discovered using HOP.

The aims of this study were to identify volatiles in *Streptomyces* with antifungal activity and to describe their mechanism(s) of action.

## 2. Results

### 2.1. Antifungal volatiles emitted by *Streptomyces* spp.

**Figure 1.**
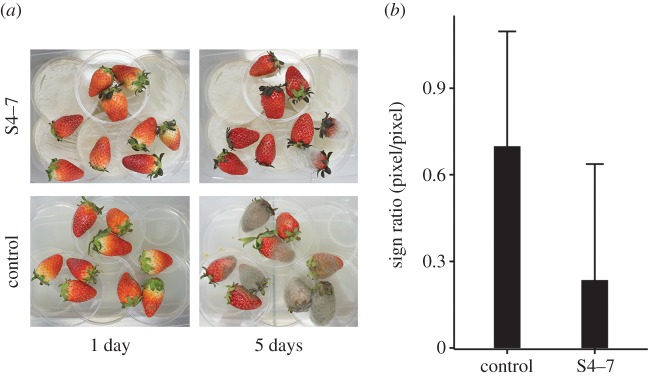
Control of fungal infection of strawberries by S4–7 volatiles. This test was conducted at room temperature. (*a*) *Botrytis cinerea* colonization of strawberry; (*b*) Visualization of *B. cinerea* sign ratio. Error bars indicate standard deviations. Berries had less coverage of *B. cinerea* mycelia and spores in the presence of the S4–7 volatile than in the control (*p* = 0.026, *t*-test).

To measure the antifungal activity of *Streptomyces* volatiles, *Botrytis cinerea*, *Colletotrichum gloeosporioides*, *Fusarium oxysporum*, *Gibberella moniliformis*, *Phytophthora nicotinae*, *Rhizoctonia cerealis* and *R. solani* were exposed to volatiles from *Streptomyces* strains S2, S4–7 and S8 (electronic supplementary material, figure S1). Mycelial growth of *B. cinerea*, *C. gloeosporioides, P. nicotinae* and *R. solani* was clearly inhibited by the S4–7 and S8 volatiles. Grey mould disease, caused by *B. cinerea,* of strawberry was significantly reduced when berries were stored with S4–7 cultured plates at room temperature (figure 1). With the treatment of strain S4–7, *B. cinerea* disease sign ratio was 0.30 ± 0.39, while that of the control was 0.76 ± 0.40 (*p* = 0.026, *t*-test).

### 2.2. Analysis of *Streptomyces* spp. volatiles' main components

The volatiles produced by strains S2, S4–7 and S8 were identified by solid phase micro-extraction (SPME) and gas chromatography–mass spectrometry (GC-MS) analysis. The volatiles of S4–7 and S8 contain three main compounds with the same retention times (RTs)—14.2, 16.4 and 17.0 min, respectively. However, S2 produced only one volatile in common with strains S4–7 and S8 (RT 14.2 min) (electronic supplementary material, figure S2). Mass spectra of the three compounds matched geosmin (RT 14.2 min), caryolan-1-ol (RT 16.4 min) and torreyol (RT 17.2 min) (electronic supplementary material, table S1) in the mass spectral library. Geosmin is produced by many *Streptomyces* spp. and has a distinct earthy odour.

**Figure 2.**
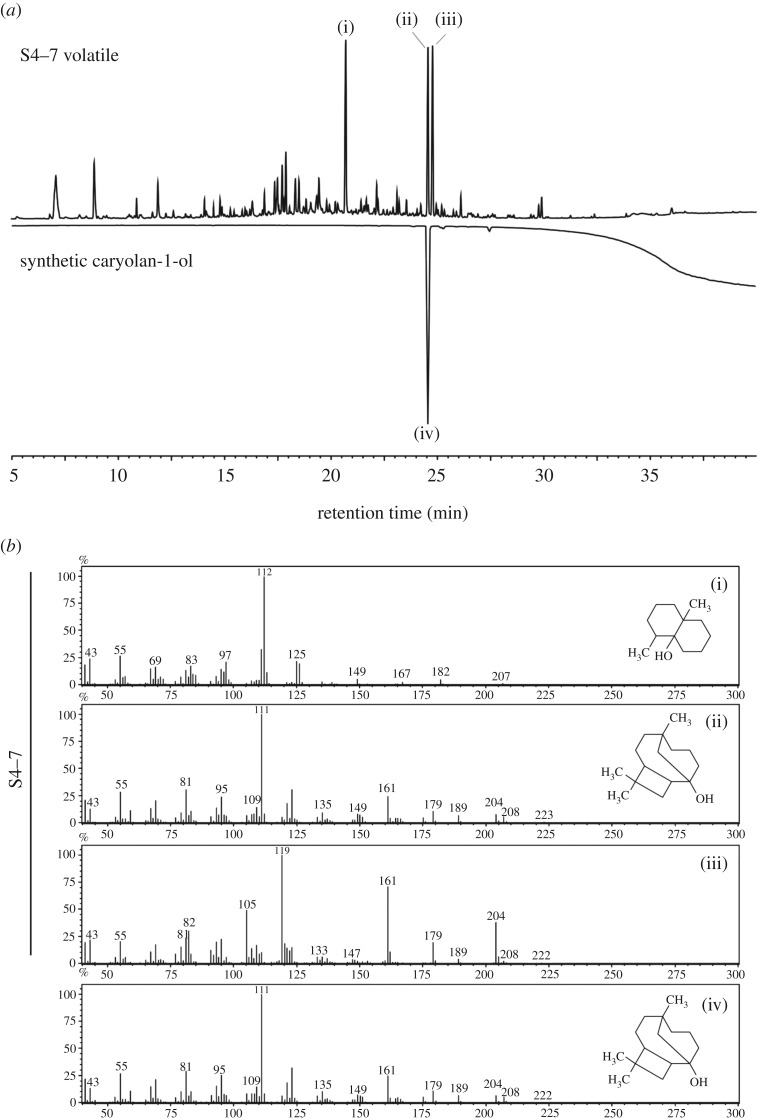
GC-MS (GC-2010 coupled with GCMS-QP2010 plus) analysis of S4–7 volatiles and synthetic caryolan-1-ol. (i) Geosmin; (ii and iv) caryolan-1-ol; (iii) unknown sesquiterpene. (*a*) GC of total ion current of synthetic caryolan-1-ol. S4–7 volatile and synthetic caryolan-1-ol have identical retention times (24 min). (*b*) Mass spectra comparison of S4–7 volatiles and synthetic caryolan-1-ol. Synthetic caryolan-1-ol (iv) mass spectrum coincides with S4–7 caryolan-1-ol (ii).

The main volatile components were predicted to be sesquiterpenes due to the three-isoprene terpene units in their chemical structures. Analysis of the KEGG database showed that S4–7 had a sesquiterpene synthetic pathway through the MEP/DOXY pathway (electronic supplementary material, figure S3). By antiSMASH, terpene synthesis gene clusters were predicted in S4–7 and S8 (GenBank Accession: GCA_000932225.1 for S4–7, CP015362 for S8). Geosmin synthase, caryolan-1-ol synthase and putative sesquiterpene cyclase were selected (table 1). *Streptomyces griseus* has been reported to produce caryolan-1-ol [19]. The GC-MS retention times (figure 2*a*) and mass spectra patterns (figure 2*b*) of naturally produced caryolan-1-ol and synthesized caryolan-1-ol were identical.

**Table 1.** Secondary metabolism analysis of *Streptomyces* using antiSMASH.

strain	antiSMASH			Blastp			
	similar known terpene cluster	gene similarity (%)	size (bp)	terpene-related enzyme	total score	ident (%)	accession
S4–7	hopene biosynthesis	69	26 574	squalene-hopene cyclase	1347	100	WP044 373 054.1
				geranylgeranyl diphosphate synthase	724	99	SCF60437.1
				squalene synthase HpnD	630	99	EWS95805.1
				squalene synthase HpnC	592	99	WP 030800744.1
	—	—	21 056	(+)-caryolan-1-ol synthase	681	99	SBU91888.1
	isorenieratene biosynthesis	85	26 117	lycopene beta-cyclase	813	100	KIX34058.1
				phytoene synthase	694	100	WP 030811369.1
				geranylgeranyl pyrophosphate synthetase	707	100	KIX34053.1
	—	—	66 745	*geosmin synthase*	1509	100	WP 044369842.1
	isorenieratene biosynthesis	85	26 327	geranylgeranyl pyrophosphate synthase	753	99	WP 044373694.1
				phytoene synthase	664	100	WP 044373656.1
				lycopene cyclase	783	100	WP 044373647.1
S8	isorenieratene biosynthesis	100	26 022	geranylgeranyl diphosphate synthase	822	100	SCK22258.1
				phytoene synthase	652	99	SCK22276.1
				lycopene beta-cyclase	780	99	SCK22321.1
	hopene biosynthesis	69	26 574	squalene-hopene cyclase	1342	99	WP 028418748.1
				geranylgeranyl diphosphate synthase,	725	99	SCD75724.1
				squalene synthase HpnD	624	99	WP 028418746.1
				squalene synthase HpnC	594	99	SCK56037.1
	—	—	21 056	(+)-*caryolan-1-ol synthase*	658	99	SCK14242.1
	—	—	22 214	*geosmin synthase*	1503	99	SCK19404.1

### 2.3. Antifungal activity of caryolan-1-ol

**Figure 3.**
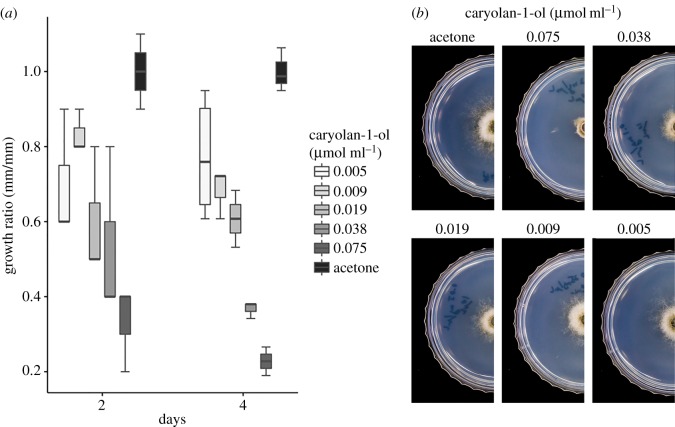
Growth inhibition of *B. cinerea* by synthetic caryolan-1-ol. (*a*) Growth ratio using box-and-whisker plots. (*b*) Growth of *B. cinerea* at 4 days. Acetone (10 μl) was used as a control treatment. Synthetic caryolan-1-ol inhibited the growth and spore formation.

Geosmin is known to have no antibiotic activity [20], but no information is available about the bioactivity of caryolan-1-ol. The collected mixture A, with caryolan-1-ol and the unknown sesquiterpene (electronic supplementary material, table S2) as main constituents (electronic supplementary material, figure S4), inhibited *B. cinerea* mycelial growth at 20 and 40 µl of mixture A. Synthetic caryolan-1-ol (0.005–0.075 µmol ml^−1^) inhibited mycelial growth in a dose-dependent manner (figure 3). Half maximal inhibitory concentration (IC_50_) of the synthetic caryolan-1-ol was 0.026 µmol ml^−1^ for *B. cinerea* after 4 days of exposure.

### 2.4. Mode of action of caryolan-1-ol antifungal activity

**Figure 4.**
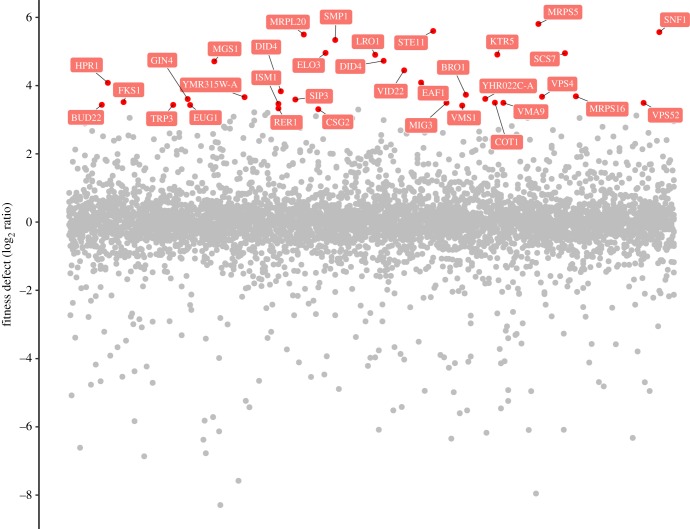
Genes affected by caryolan-1-ol. Thirty-three deletion strains with a fitness defect score of 3.3 or greater were selected.

For HOP assay, growth of the diploid strain *S. cerevisiae* BY4743 was measured with different concentrations of caryolan-1-ol (electronic supplementary material, figure S5). *Saccharomyces cerevisiae* growth was reduced by 4.28%, 11.80% and 30.80% by 0.063, 0.125 and 0.250 µmol ml^−1^ caryolan-1-ol, respectively. A threshold for the HOP screen with caryolan-1-ol (0.188 µmol ml^−1^) was defined with a fitness defect of 3.3 or greater (figure 4). Based on these criteria, caryolan-1-ol affected 33 deletion strains (electronic supplementary material, table S3). Based on Gene Ontology (GO) enrichment, caryolan-1-ol affected the following biological functions: lipid synthesis (*sur4, scs7* and *lro1*) associated with ceramide; chromatin remodelling (*eaf1* and *hpr1*); osmotic stress response (*smp1* and *ste11*); DNA replication stress response (*ste11, mgs1, cot1* and *csg2*); and transported organelles related to endosomes, vesicles, vacuoles; Golgi and endoplasmic reticulum (*did4, vid22, vps4, sip3, cot1, vma9, vps52, eug1, vms1, rer1* and *csg2*).

**Figure 5.**
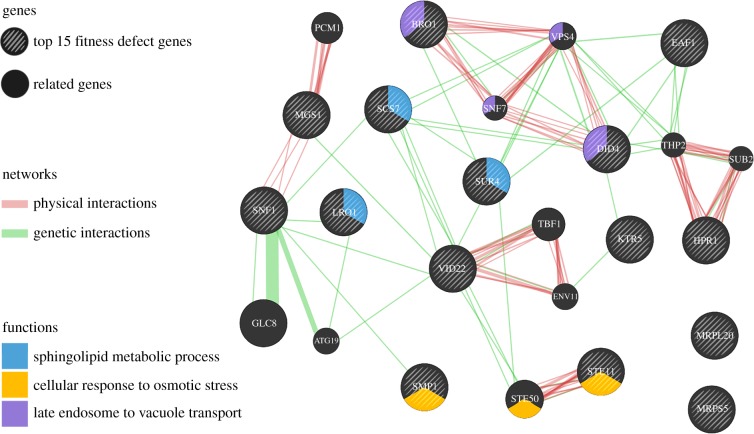
Network analysis of high defect score for the top 15 genes. Visualization was perfomed by GeneMANIA (http://genemania.org). Genes are functionally grouped into sphingolipid, cellular response to omotic stress and late endosome to vacuole transport.

**Figure 6.**
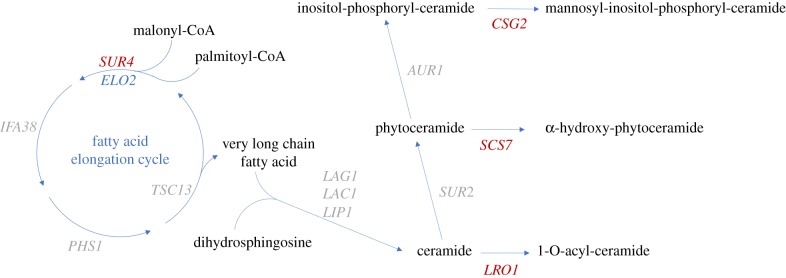
Caryolan-1-ol affects very long chain fatty acid and ceramide-based lipid biosynthesis pathways. The illustration shows the sphingolipid synthesis pathway in yeast. Blue lines show synthetic pathway and red letters indicate genes with high fitness defect score (greater than 3.3) in figure 4.

Using network analysis and GO provided by the Saccharomyces Genome Database (SGD), the 15 most sensitive deletion strains corresponded to genes associated with the sphingolipid metabolic process (*sur4, scs7, lro1*), cellular response to osmotic stress (*smp1, ste11*) and late endosome vacuole transport (*bro1, did4*) (figures 5 and 6). Comprehensive GO process results were categorized in the electronic supplementary material, figure S6. The GO component ‘Endomembrane system’ was shared by 14 of the 33 significantly sensitive strains (table 2). Collectively, these observations suggested that caryolan-1-ol affects fungal growth and probably acts by affecting membrane lipid processes and the intercellular transport system in fungi (figure 6).

**Table 2.** GO term analysis of the high fitness defect score genes in HOP assay (*p* > 0.05). FDR, false discovery rate; EFP, expected false positives.

Gene Ontology term		cluster frequency	background frequency	*p*-value	FDR	EFP	genes annotated to the term
component	endomembrane system	14/33 genes (42.4%)	978/7165 background genes (13.6%)	0.004	0.00%	0.00	CSG2, RER1, VMS1, SNF1, VPS52, EUG1, DID4, SUR4, SCS7, KTR5, SIP3, LRO1, BRO1, VPS4
process	intralumenal vesicle formation	3/33 genes (9.1%)	7/7165 background genes (0.1%)	0.001	0.00%	0.00	DID4, BRO1, VPS4
	endosome organization	3/33 genes (9.1%)	16/7165 background genes (0.2%)	0.012	0.02%	0.04	DID4, BRO1, VPS4
	maintenance of location	4/33 genes (12.1%)	45/7165 background genes (0.6%)	0.013	0.01%	0.04	RER1, DID4, LRO1, VPS4
	sphingolipid metabolic process	4/33 genes (12.1%)	45/7165 background genes (0.6%)	0.013	0.01%	0.04	CSG2, SUR4, SCS7, LRO1
	biological regulation	18/33 genes (54.5%)	1597/7165 background genes (22.3%)	0.014	0.01%	0.04	CSG2, SMP1, RER1, VMA9, HPR1, SNF1, VPS52, GIN4, MIG3, DID4, FKS1, STE11, MGS1, SIP3, LRO1, COT1, BRO1, VPS4
	endosomal transport	5/33 genes (15.2%)	102/7165 background genes (1.4%)	0.024	0.02%	0.10	VPS52, DID4, SUR4, BRO1, VPS4
	regulation of biological quality	9/33 genes (27.3%)	429/7165 background genes (6.0%)	0.025	0.01%	0.10	CSG2, RER1, VMA9, VPS52, DID4, FKS1, LRO1, COT1, VPS4
	late endosome to vacuole transport	4/33 genes (12.1%)	55/7165 background genes (0.8%)	0.028	0.01%	0.10	DID4, SUR4, BRO1, VPS4
	membrane budding	3/33 genes (9.1%)	22/7165 background genes (0.3%)	0.034	0.02%	0.14	DID4, BRO1, VPS4

## 3. Discussion

This study was designed to characterize antifungal volatiles in *Streptomyces*, to identify new antifungal volatile components and to analyse the genetic effects of these volatile components on fungi. The antifungal test demonstrated that *Streptomyces* produces antifungal volatiles against a variety of phytopathogenic fungi in different classes. Hora & Baker [14] reported that volatiles of certain *Streptomyces* strains inhibited growth of *Trichoderma viride*, *Zygorhynchus vuilleminii*, *Gonatobotrys simplex* and *Cladosporium* sp. The grey mould pathogen *B. cinerea* is visibly ashy when spores are generated. However, the pathogen on strawberry that was exposed to S4–7 volatiles did not form spores and its growth was inhibited. GC-MS analysis using strains S2, S4–7 and S8 indicated that the three major components are terpenes: geosmin, an unknown sesquiterpene and caryolan-1-ol. In the secondary metabolism analysis of the genome, three terpene synthetic clusters corresponding to the GC-MS analyses were also confirmed. These results indicate that caryolan-1-ol is produced by strains S4–7 and S8; the GC-MS results comparing S4–7 volatiles and synthetic caryolan-1-ol were an identical match. Our results support previous studies that showed that caryolan-1-ol was released by specific *Streptomyces* strains*,* including *S. griseus* [19,21]. In addition, caryolan-1-ol has been found in several plants and fungi [22–28]. These types of caryolan-1-ol can consist of 32 enantiomers, but only three have been documented: (+)-caryolan-1-ol (*S. griseus*), (−)-caryolan-1-ol (*Zingiber officinale* and *Hardwickia pinnata*) and 1R-caryolan-1-ol (*Aspergillus niger*). Since the S4–7 and S8 genomes have (+)-caryolan-1-ol synthase, they may produce only (+)-caryolan-1-ol.

Although the synthesized caryolan-1-ol has not been characterized as a type of optical isomer, *B. cinerea* growth was inhibited by the isolated and the synthesized caryolan-1-ol. These similar antifungal activities between the *Streptomyces* mixture of volatiles and the synthesized caryolan-1-ol indicated that the antifungal activity was caused by the caryolan-1-ol. Inhibition by caryolan-1-ol coincided well with an earlier report on unknown volatiles from *S. griseus* affecting fungal growth and sporulation of *Gloeosporium aridum* [12].

To investigate the biological functions of caryolan-1-ol, the screening of affected genes was conducted by HOP assay. It was shown that 33 homozygous deletion strains were susceptible to caryolan-1-ol with a fitness defect score of 3.3 or higher. Fourteen of the 33 strains were deleted for genes associated with the endomembrane system, whose role it is to separate the structural compartments or organelles, including the nuclear envelope, endoplasmic reticulum, Golgi apparatus, vacuoles, vesicles, Spitzenkörper and plasma membrane. The fitness defects in the top 15 strains were related to sphingolipid metabolic processes, response of osmotic stress and late endosome to vacuole transport. The cellular process results suggested that these genes were related to sphingolipid synthesis, vesicle transport, vesicle trafficking and maintenance.

Sphingolipids, which are classified as ceramides, sphingomyelins and glycosphingolipids, are especially important because they constitute 40% of plasma membranes in yeast [29,30]. This supports our observation from the HOP results that caryolan-1-ol inhibited the sensitivity strains that were deleted for osmotic stress response genes (*smp1* and *ste11*). It has been documented that sphingolipids are involved in numerous cellular processes including cell senescence, cell differentiation, apoptosis, cell-cycle arrest, cell proliferation, etc. [31]. *sur4* (*elo3*) synthesizes a ceramide molecule which is the backbone of sphingolipids [32,33]. Trajkovic *et al*. [34] reported that ceramide is required for budding exosome into multi-vesicular endosome formation in fungi. The selected *scs7* and *csg2* are known to synthesize α-hydroxy-phytoceramide and mannosyl-inositol-phosphoryl-ceramide (MIPC), which are made from ceramide and phytoceramide [33,35]. The MIPC is required for proper localization of plasma membrane proteins using vesicle trafficking as well as maintenance of cell morphology and vacuoles [36]. Based on these previous studies, caryolan-1-ol may also interrupt other GO processes such as vesicle transport, vesicle trafficking and maintenance of location that are linked to sphingolipid metabolism. The vesicle supply at the fungal hypha tip and Spitzenkörper are key components of the endomembrane system. *Botrytis cinerea* growth inhibition probably occurred due to fungal Spitzenkörper damage.

In conclusion, this is the first report on the biological role of caryolan-1-o1, a volatile produced by *Streptomyces*. It has antifungal properties that are caused by perturbation of the endomembrane system in fungi. Further work will help dissect the mechanism by which this volatile affects sphingolipid metabolism, vesicle formation, vesicle trafficking and membrane localization.

## 4. Material and methods

### 4.1. Antifungal test of *Streptomyces* volatiles

*Streptomyces* sp. strain S2, *Streptomyces* sp. strain S8, and *S. griseus* strain S4–7 were isolated from the rhizosphere of a 15-year old strawberry field [37]. *Streptomyces* sp. strains S2 and S8 were isolated from the turfgrass rhizosphere [38].

*Streptomyces* spp. were cultured for 10 days at 27°C in potato dextrose peptone (PDK) agar media (per 1 l: potato dextrose: 10 g; peptone: 10 g; agar: 20 g) in 90 × 15 mm Petri dishes. *Botrytis cinerea*, *C. gloeosporioides*, *F. oxysporum*, *G. moniliformis*, *P. nicotinae*, *R. cerealis* and *R. solani* were inoculated on PDK agar media in 90 × 15 mm Petri dishes. To test the antifungal activity of the volatiles of *Streptomyces* spp., fungal plates and *Streptomyces* spp. plates without the lids were put into a 2.7 l airtight container and incubated at 27°C. Fungal mycelial growth was measured every day in the same container with four replicates for each treatment.

### 4.2. Test for controlling postharvest disease by *Streptomyces* volatiles

Nine strawberries and six 5-day old S4–7 PDK plates were in the airtight containers. The PDK plates were located in the bottom of the container and the strawberries were put on top of the PDK plates. The containers were incubated at room temperature for 5 days. The pixels with no signs were counted using a quick selection tool and histogram bar window to display the number of pixels in Photoshop CS5. The sign ratio could be described using the following equation:
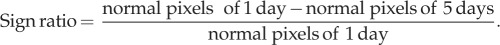
The statistical analyses of sign ratios were performed using the Shapiro–Wilk's normality test, Bartlett test of homogeneity of variances and two sample *t*-tests using R program v. 3.2.3.

### 4.3. Gas chromatography–mass spectrometry

*Streptomyces* spp. were cultured at 27°C for 10 days in PDK agar media in 60 × 15 mm Petri dishes. The Petri dishes containing the *Streptomyces* cultures were put into a glass jar, and the volatiles from the *Streptomyces* spp. were collected using SPME at 80°C for 30 min. The fibres of SPME were composed of divinylbenzene/carboxen/polydimethylsiloxane. The volatiles collected from the SPME fibres were analysed by electron impact gas chromatography–mass spectrometry (EI-GC-MS; GC2010 plus-GCMS-TQ8030; Shimadzu, Tokyo, Japan) equipped with a Rtx-5MS column (30 m × 0.25 mm i.d.×0.25 µm film thickness, Restek Co., PA, USA). The carrier gas was programmed as 1.1 ml min^−1^ for an initial 2 min, and then gradually decreased to 0.5 ml min^−1^ for 20 min and held there. The oven temperature was maintained at 50°C for 2 min, and then increased to 250°C at 10°C min^−1^ with that temperature being held for 8 min. The temperatures of the interface and ion source were set at 230 and 280°C, respectively.

Volatiles collected from *Streptomyces* spp. and the synthesized caryolan-1-ol was analysed by GC-MS (GC-2010 coupled with GCMS-QP2010 plus; Shimadzu, Tokyo, Japan) using HP-Innowax (30 m × 0.25 mm × 0.25 µm film thickness; J&W Scientific). The oven temperature was maintained at 40°C for 1 min, then raised to 250°C at 6°C min^−1^ and held for 4 min. Helium was the carrier gas at a flow rate of 1.0 ml min^−1^.

### 4.4. Secondary metabolism pathway analysis of the S4–7 and S8 genomes

The S4–7 and the S8 genomic sequences were examined by CLgenomics (v. 1.53; ChunLab, Seoul, South Korea), which supports the secondary metabolism pathway analysis using the KEGG database. Gene cluster analysis used the secondary metabolism finder tool antiSMASH (https://antismash.secondarymetabolites.org/).

### 4.5. Mass collection of volatiles of *Streptomyces griseus* S4–7

*Streptomyces griseus* S4–7 was cultured for 10 days at 27°C in PDK agar media in 90 × 15 mm Petri dishes. To collect a large amount of the *S. griseus* S4–7 volatile compounds, 20 Petri dishes were inoculated with *S. griseus* S4–7 and then put into a container (6 l). The volatiles were collected with an absorbent, Super Q 100 (approx. 100 mg, 2 cm in length, 6 mm OD in a glass tube; Alltech, Deerfield, Illinois, USA), connected to an air pump. Charcoal-filtered air was introduced into the container. The container was aerated at a rate of 600 ml min^−1^. The volatiles were collected for 10 days. The experiment was replicated three times with 20 new Petri dishes inoculated with *S. griseus* S4–7. The captured volatiles were eluted with 2 ml of hexane. The eluate was concentrated to approximately 20 μl and subjected to chromatography (1 g; Wakogel-200, Wako, Osaka, Japan) using hexane and diethyl ether as eluents. In the diethyl ether fraction (1 mg), caryolan-1-ol (13.9%) and an unknown sesquiterpene (23.3%) were the major constituents (table 1). This fraction was used for bioassays.

### 4.6. Synthesis of caryolan-1-ol

Caryolan-1-ol was synthesized by modifying the method of Iwamuro *et al.* [39]. β-Caryophyllene (3.0 g; 15 mmol, TCI, Tokyo, Japan) was dissolved in acetic acid (100 ml) with BHT (butylated hydroxytoluene; Sigma-Aldrich). The solution was passed twice through a column packed with Amberlyst 15 (Alfa Aesar, Lancaster, UK) at a rate of 2–3 ml min^−1^. The solution was diluted with ether and then washed five times with 2N NaOH, followed by washing with water and brine, and dried. After removing the solvent, the residue was subjected to silica gel column chromatography (Wakogel-200) to isolate the caryolan-1-ol (45% yield, 53% purity). Further purification using a fraction collector was done and 94.0% pure caryolan-1-ol (100 mg) was obtained. The caryolan-1-ol was confirmed via nuclear magnetic resonance (NMR) and GC-MS. NMR spectra agreed with those published in previous reports [40,41]. ^1^H and ^13^C NMR (500 and 125 MHz, respectively) analysis was conducted with a Bruker DRX-500 spectrometer using TMS in CDCl_3_ as an internal standard at GNU's Center for Research Facilities.

^1^H NMR (500 MHz, CDCl_3_) *δ* (ppm): 2.241 (q, *J* = 10.0, 1H), 1.851–1.796 (m, 1H), 1.770–1.646 (m, 3H), 1.627–1.587 (m, 1H), 1.553–1.510 (m, 2H), 1.497–1.431 (m, 2H), 1.399–1.275 (m, 3H), 1.051–1.024 (m, 2H), 1.010 (s, 3H), 1.005 (s, 3H), 0.886 (s, 3H). ^13^C NMR (126 MHz, CDCl3) *δ* (ppm): 70.99, 48.76, 44.78, 39.52, 38.62, 37.46, 36.63, 34.98, 34.80, 34.42, 33.24, 30.54, 21.91, 20.87, 20.82.

### 4.7. Antifungal test of collected volatiles and synthetic caryolan-1-ol

To test antifungal activity, the volatile mixture concentration of S4–7 was prepared at 5 µg µl^−1^ and 40, 20 and 10 μl samples used. The synthetic caryolan-1-ol was diluted to 0.075, 0.038, 0.019, 0.009 and 0.005 μmol ml^−1^. A paper disc (8 mm) treated with a fraction of volatiles mixture or synthesized caryolan-1-ol was put in the lid of the Petri dish cover and *B. cinerea* was grown on the agar at 27°C. After 4 days of incubation, mycelial growth was measured.

### 4.8. Homozygous profiling assay

To discover drug effects, the IC_20_ is generally used for the HOP assay [42]. The caryolan-1-ol IC_20_ was determined by the fallowing approach. *S. cerevisiae* BY4743 was inoculated in yeast extract peptone dextrose (YPD; 10 g yeast extract, 20 g peptone, 20 g dextrose per l) broth in a 24-well plate. Synthesized caryolan-1-ol was added at 250, 125 and 62.5 nmol. Growth of BY4741 was measured by absorbance at 600 nm every 15 min for 24 h at 30°C. The IC_20_ was calculated to be 188 nmol ml^−1^. The homozygous diploid deletion strains pool (approx. 4800 strains) was inoculated in the caryolan-1-ol (188 nmol ml^−1^) amended and non-amended YPD broth at 30°C for 24 h. Total genomic DNA of the pools was extracted using the YeaStar™ Genomic DNA kit (Zymo Research Co, USA) and its uptags and downtags were amplified by PCR. Amplification quantification was performed with Affymetrix TAG4 arrays [42]. Caryolan-1-ol sensitive strains were selected using fitness defect score expressed as the log 2 ratios. Deletion genes of these strains were analysed by GeneMANIA (http://genemania.org), Saccharomyces Genome Database (SGD) Gene Ontology (GO) term finder and Blast2GO (v. 4.0.7).

## Supplementary Material

Supplementary tables and figures

## Data accessibility

Additional data are available in the electronic supplementary material.

## References

[1] Hopwood DA 2007 Streptomyces in nature and medicine: The antibiotic makers. Oxford, UK: Oxford University Press.

[2] Omura S et al. 2001 Genome sequence of an industrial microorganism *Streptomyces avermitilis*: deducing the ability of producing secondary metabolites. Proc. Natl Acad. Sci. USA 98, 12 215–12 220. (doi:10.1073/pnas.211433198) 10.1073/pnas.211433198 PMC59794 11572948

[3] Khan ST, Komaki H, Motohashi K, Kozone I, Mukai A, Takagi M, Shin-ya K 2011 *Streptomyces* associated with a marine sponge *Haliclona* sp.; biosynthetic genes for secondary metabolites and products. Environ. Microbiol. 13, 391–403. (doi:10.1111/j.1462-2920.2010.02337.x) 20849448 10.1111/j.1462-2920.2010.02337.x

[4] Patzer SI, Braun V 2010 Gene cluster involved in the biosynthesis of griseobactin, a catechol-peptide siderophore of *Streptomyces* sp. ATCC 700974. J. Bacteriol. 192, 426–435. (doi:10.1128/JB.01250-09) 19915026 10.1128/JB.01250-09 PMC2805312

[5] Bérdy J 2012 Thoughts and facts about antibiotics: where we are now and where we are heading. J. Antibiot. 65, 385–395. (doi:10.1038/ja.2012.27) 22511224 10.1038/ja.2012.27

[6] Hopwood DA 2006 Soil to genomics: the *Streptomyces* chromosome. Annu. Rev. Genet. 40, 1–23. (doi:10.1146/annurev.genet.40.110405.090639) 16761950 10.1146/annurev.genet.40.110405.090639

[7] Pieculla B, Degenhardt J 2014 The emerging importance of microbial volatile organic compounds. Plant Cell Environ. 37, 811–812. (doi:10.1111/pce.12254) 24329873 10.1111/pce.12254

[8] Schulz S, Dickschat JS 2007 Bacterial volatiles: the smell of small organisms. Nat. Prod. Rep. 24, 814–842. (doi:10.1039/b507392h) 17653361 10.1039/b507392h

[9] Peñuelas J, Asensio D, Tholl D, Wenke K, Rosenkranz M, Piechulla B, Schnitzler JP 2014 Biogenic volatile emissions from the soil. Plant Cell Environ. 37, 1866–1891. (doi:10.1111/pce.12340) 24689847 10.1111/pce.12340

[10] Schöller CE, Gürtler H, Pedersen R, Molin S, Wilkins K 2002 Volatile metabolites from actinomycetes. J. Agric. Food Chem. 50, 2615–2621. (doi:10.1021/jf0116754) 11958631 10.1021/jf0116754

[11] Kai M, Haustein M, Molina F, Petri A, Scholz B, Piechulla B 2009 Bacterial volatiles and their action potential. Appl. Microbiol. Biotechnol. 81, 1001–1012. (doi:10.1007/s00253-008-1760-3) 19020812 10.1007/s00253-008-1760-3

[12] McCain AH 1966 A volatile antibiotic produced by *Streptomyces griseus*. Phytopathology 5, 150.

[13] Whaley JW, Boyle AM 1967 Antibiotic production by *Streptomyces* species from the rhizosphere of desert plants. Phytopathology 57, 347–351. 6039498

[14] Hora TS, Baker R 1972 Soil fungistasis: microflora producing a volatile inhibitor. Trans. Br. Mycol. Soc. 59, 491–500. (doi:10.1016/S0007-1536(72)80130-X)

[15] Fries N 1973 Effects of volatile organic compounds on the growth and development of fungi. Trans. Br. Mycol. Soc. 60, 1–21. (doi:10.1016/S0007-1536(73)80055-5)

[16] Ericson E, Hoon S, Onge RPS, Giaever G, Nislow C 2010 Exploring gene function and drug action using chemogenomic dosage assays. Methods Enzymol. 470, 233–255. (doi:10.1016/S0076-6879(10)70010-0) 20946813 10.1016/S0076-6879(10)70010-0

[17] Parsons AB et al. 2004 Integration of chemical-genetic and genetic interaction data links bioactive compounds to cellular target pathways. Nat. Biotechnol. 22, 62–69. (doi:10.1038/nbt919) 14661025 10.1038/nbt919

[18] Chang M, Bellaoui M, Boone C, Brown GW 2002 A genome-wide screen for methyl methanesulfonate-sensitive mutants reveals genes required for S phase progression in the presence of DNA damage. Proc. Natl Acad. Sci. USA 99, 16 934–16 939. (doi:10.1073/pnas.262669299) 10.1073/pnas.262669299 PMC139247 12482937

[19] Nakano C, Horinouchi S, Ohnishi Y 2011 Characterization of a novel sesquiterpene cyclase involved in (+)-caryolan-1-ol biosynthesis in *Streptomyces griseus*. J. Biol. Chem. 286, 27 980–27 987. (doi:10.1074/jbc.M111.265652) 10.1074/jbc.M111.265652 PMC3151043 21693706

[20] Seipke RF, Kaltenpoth M, Hutchings MI 2012 *Streptomyces* as symbionts: an emerging and widespread theme? FEMS Microbial. Rev. 36, 862–876. (doi:10.1111/j.1574-6976.2011.00313.x) 10.1111/j.1574-6976.2011.00313.x 22091965

[21] Citron CA, Barra L, Wink J, Dickschat JS 2015 Volatiles from nineteen recently genome sequenced actinomycetes. Org. Biomol. Chem. 13, 2673–2683. (doi:10.1039/C4OB02609H) 25585196 10.1039/c4ob02609h

[22] Gherib M, Atik Bekkara F, Bekhechi C, Bighelli A, Casanova J, Tomi F 2014 Composition and antimicrobial activity of the essential oil from Algerian *Warionia saharae* Benth. & Hook. J. Essent. Oil Res. 26, 385–391. (doi:10.1080/10412905.2014.937008)

[23] Koo HJ, Gang DR 2012 Suites of terpene synthases explain differential terpenoid production in ginger and turmeric tissues. PLoS ONE, 7, e51481 (doi:10.1371/journal.pone.0051481) 23272109 10.1371/journal.pone.0051481 PMC3525583

[24] Tressl R, Engel KH, Kossa M, Koeppler H 1983 Characterization of tricyclic sesquiterpenes in hop (*Humulus lupulus* var. Hersbrucker Spaet). J. Agric. Food Chem. 31, 892–897. (doi:10.1021/jf00118a056)

[25] Meekijjaroenroj A, Bessière JM, Anstett MC 2007 Chemistry of floral scents in four *Licuala* species (Arecaceae). Flavour Fragr. J. 22, 300–310. (doi:10.1002/ffj.1797)

[26] Misra R, Pandey RC, Dev S 1979 Higher isoprenoids—VIII: diterpenoids from the oleoresin of *Hardwickia pinnata* part 1: hardwickiic acid. Tetrahedron 35, 2301–2310. (doi:10.1016/0040-4020(79)80125-8)

[27] Kamatou GPP, Van Zyla RL, Van Vuurena SF, Figueiredob AC, Barrosob JG, Pedrob LG, Viljoenc AM 2008 Seasonal variation in essential oil composition, oil toxicity and the biological activity of solvent extracts of three South African *Salvia* species. S. Afr. J. Bot. 74, 230–237. (doi:10.1016/j.sajb.2007.08.002)

[28] Lamare V, Archelas A, Faure R, Ceasrio M, Pascard C, Furstoss R 1989 Microbial transformations. 14. Regioselective hydroxylation of (1R)-caryolanol by *Aspergillus niger*. A reexamination of the ^13^C NMR spectrum of caryolanol. Tetrahedron 45, 3761–3768. (doi:10.1016/S0040-4020(01)89237-1)

[29] Dickson RC, Wells GB, Schmidt A, Lester RL 1990 Isolation of mutant *Saccharomyces cerevisiae* strains that survive without sphingolipids. Mol. Cell. Biol. 10, 2176–2181. (doi:10.1128/MCB.10.5.2176) 2183021 10.1128/mcb.10.5.2176-2181.1990 PMC360565

[30] Dickson RC, Lester RL 1999 Yeast sphingolipids. Biochim. Biophys. Acta 1426, 347–357. (doi:10.1016/S0304-4165(98)00135-4) 9878820 10.1016/s0304-4165(98)00135-4

[31] Hannun YA, Obeid LM 2008 Principles of bioactive lipid signaling: lessons from sphingolipids. Nat. Rev. Mol. Cell Biol. 9, 139–150. (doi:10.1038/nrm2329) 18216770 10.1038/nrm2329

[32] Oh CS, Toke DA, Mandala S, Martin CE 1997 ELO2 and ELO3, homologues of the *Saccharomyces cerevisiae* ELO1 gene, function in fatty acid elongation and are required for sphingolipid formation. J. Biol. Chem. 272, 17 376–17 384. (doi:10.1074/jbc.272.28.17376) 9211877 10.1074/jbc.272.28.17376

[33] Dickson RC, Lester RL 2002 Sphingolipid functions in *Saccharomyces cerevisiae*. Biochim. Biophys. Acta 1583, 13–25. (doi:10.1016/S1388-1981(02)00210-X) 12069845 10.1016/s1388-1981(02)00210-x

[34] Trajkovic K, Hsu C, Chiantia S, Rajendran L, Wenzel D, Wieland F, Schwille P, Brügger B, Simons M 2008 Ceramide triggers budding of exosome vesicles into multivesicular endosomes. Science 319, 1244–1247. (doi:10.1126/science.1153124) 18309083 10.1126/science.1153124

[35] Dunn TM, Haak D, Monaghan E, Beeler TJ 1998 Synthesis of monohydroxylated inositolphosphorylceramide (IPC-C) in *Saccharomyces cerevisiae* requires Scs7p, a protein with both a cytochrome b5 like domain and a hydroxylase/desaturase domain. Yeast 14, 311–321. (doi:10.1002/(SICI)1097-0061(19980315)14:4<311::AID-YEA220>3.0.CO;2-B) 9559540 10.1002/(SICI)1097-0061(19980315)14:4<311::AID-YEA220>3.0.CO;2-B

[36] Nakase M, Tani M, Morita T, Kitamoto HK, Kashiwazaki J, Nakamura T, Hosomi A, Tanaka N, Takegawa K 2010 Mannosylinositol phosphorylceramide is a major sphingolipid component and is required for proper localization of plasma-membrane proteins in *Schizosaccharomyces pombe*. J. Cell. Sci. 123, 1578–1587. (doi:10.1242/jcs.059139) 20388730 10.1242/jcs.059139

[37] Cha JY et al. 2016 Microbial and biochemical basis of a Fusarium wilt-suppressive soil. ISME J. 10, 119–129. (doi:10.1038/ismej.2015.95) 26057845 10.1038/ismej.2015.95 PMC4681868

[38] Lee JH, Min GY, Shim GY, Jeon CW, Kwak YS 2015 Physiological characteristics of actinomycetes isolated from turfgrass rhizosphere. Weed Turf. Sci. 4, 348–359. (doi:10.5660/WTS.2015.4.4.348)

[39] Iwamuro H, Shieh B, Takenokuchi H, Matsubara Y 1982 Hydration products of nine sesquiterpene hydrocarbons. J. Oleo Sci. 31, 110–112. (doi:10.5650/jos1956.31.110)

[40] da Silva Rocha KA, Rodrigues NV, Kozhevnikov IV, Gusevskaya EV 2010 Heteropoly acid catalysts in the valorization of the essential oils: acetoxylation of β-caryophyllene. Appl. Catal. A Gen. 374, 87–94. (doi:10.1016/j.apcata.2009.11.032)

[41] Yang G, Wu P, Zhou Z, He X, Meng W, Zhang Z 2012 Direct hydration of β-caryophyllene. Ind. Eng. Chem. Res. 51, 15 864–15 871. (doi:10.1021/ie301294f)

[42] Smith AM, Durbic T, Oh J, Urbanus M, Proctor M, Heisler LE, Giaever G, Nislow C 2011 Competitive genomic screens of barcoded yeast libraries. J. Vis. Exp. 54, 2864 (doi:10.3791/2864) 10.3791/2864 PMC3211125 21860376

