# Supplementary material for: Caryolan-1-ol, an antifungal volatile produced by Streptomyces spp., inhibits the endomembrane system of fungi
Source: Open Biol. 2017 Jul 19;7(7):170075. doi: 10.1098/rsob.170075 (PMC5541347; doi:10.1098/rsob.170075)
Supplement: Supplementary tables and figures [file rsob170075supp1.docx]

**Supplementary Information: Caryolan-1-ol, an Antifungal Volatile Produced by *Streptomyces* spp., Inhibits Endomembrane System of Fungi**

Table S1. List of *Streptomyces* volatile components analyzed by mass spectrometer (EI-GC-MS; GC2010 plus-GCMS-TQ8030)

| Strain | Retention Time (min) | Area  (%) | Mass spectra  Similarity (%) | Compound name | Molecular weight | Formula | Library |
| --- | --- | --- | --- | --- | --- | --- | --- |
| S2 | 12.9 | 9.47 | 81 | 6-Methyl-cyclodec-5-enol | 168 | C_11_H_20_O | Wiley9 |
|  | 14.2 | 2.89 | 93 | Geosmin | 182 | C_12_H_22_O | Wiley9 |
|  | 17.9 | 56.32 | 95 | Heptadecane | 240 | C_17_H_36_ | Wiley9 |
| S4-7 | 14.2 | 14.73 | 93 | Geosmin | 182 | C_12_H_22_O | Wiley9 |
|  | 16.4 | 28.52 | 92 | Caryolan-1-o1 | 222 | C_15_H_26_O | NIST11 |
|  | 17.0 | 38.08 | 83 | Torreyol | 222 | C_15_H_26_O | Wiley9 |
| S8 | 14.2 | 8.45 | 94 | Geosmin | 182 | C_12_H_22_O | Wiley9 |
|  | 16.4 | 8.13 | 91 | Caryolan-1-o1 | 222 | C_15_H_26_O | NIST11 |
|  | 17.0 | 35.43 | 84 | Torreyol | 222 | C_15_H_26_O | Wiley9 |
|  | 17.1 | 10.89 | 87 | Hedycaryol | 222 | C_15_H_26_O | Wiley9 |

Table S2. List of collected volatile components produced by the S4-7

|  | Components | Ratio | Mass |
| --- | --- | --- | --- |
| mixture A | caryolan-1-ol | 13.92% | 1 mg |
|  | unknown sesquiterpene | 23.27% |  |
|  | hexanol | 5.72% |  |
|  | phenyl ethanol | 7.51% |  |

Table S3. Description of 33 high fitness defect score (>3.3) genes in HOP assay

| Gene | FD^a^ | Saccharomyces Genome Database description |
| --- | --- | --- |
| MRPS5 | 5.81 | Mitochondrial ribosomal protein of the small subunit |
| STE11 | 5.60 | Signal transducing MEK kinase; involved in pheromone response and pseudohyphal/invasive growth pathways where it phosphorylates Ste7p, and the high osmolarity response pathway, via phosphorylation of Pbs2p; regulated by Ste20p and Ste50p; protein abundance increases in response to DNA replication stress |
| SNF1 | 5.57 | AMP-activated S/T protein kinase; forms a complex with Snf4p and members of the Sip1p/Sip2p/Gal83p family; required for transcription of glucose-repressed genes, thermotolerance, sporulation, and peroxisome biogenesis; regulates nucleocytoplasmic shuttling of Hxk2p; regulates filamentous growth and acts as a non-canonical GEF, activating Arf3p during invasive growth; SUMOylation by Mms21p inhibits its function and targets Snf1p for destruction via the Slx5-Slx8 Ub ligase |
| MRPL20 | 5.50 | Mitochondrial ribosomal protein of the large subunit |
| SMP1 | 5.34 | MADS-box transcription factor involved in osmotic stress response; SMP1 has a paralog, RLM1, that arose from the whole genome duplication |
| SUR4 | 4.96 | Elongase; involved in fatty acid and sphingolipid biosynthesis; synthesizes very long chain 20-26-carbon fatty acids from C18-CoA primers; involved in regulation of sphingolipid biosynthesis; lethality of the elo2 elo3 double null mutation is functionally complemented by human ELOVL1 and weakly complemented by human ELOVL3 or ELOV7 |
| SCS7 | 4.95 | Sphingolipid alpha-hydroxylase; functions in the alpha-hydroxylation of sphingolipid-associated very long chain fatty acids, has both cytochrome b5-like and hydroxylase/desaturase domains, not essential for growth |
| KTR5 | 4.91 | Putative mannosyltransferase involved in protein glycosylation; member of the KRE2/MNT1 mannosyltransferase family; KTR5 has a paralog, KTR7, that arose from the whole genome duplication |
| LRO1 | 4.90 | Acyltransferase that catalyzes diacylglycerol esterification; one of several acyltransferases that contribute to triglyceride synthesis; Lro1p and Dga1p can O-acylate ceramides; putative homolog of human lecithin cholesterol acyltransferase |
| DID4  (up tag) | 4.73 | Class E Vps protein of the ESCRT-III complex; required for sorting of integral membrane proteins into lumenal vesicles of multivesicular bodies, and for delivery of newly synthesized vacuolar enzymes to the vacuole, involved in endocytosis |
| MGS1 | 4.71 | Protein with DNA-dependent ATPase and ssDNA annealing activities; involved in maintenance of genome; interacts functionally with DNA polymerase delta; homolog of human Werner helicase interacting protein (WHIP); forms nuclear foci upon DNA replication stress |
| VID22 | 4.45 | Glycosylated integral membrane protein localized to plasma membrane; plays a role in fructose-1,6-bisphosphatase (FBPase) degradation; involved in FBPase transport from the cytosol to Vid (vacuole import and degradation) vesicles; VID22 has a paralog, ENV11, that arose from the whole genome duplication |
| EAF1 | 4.09 | Component of the NuA4 histone acetyltransferase complex; acts as a platform for assembly of NuA4 subunits into the native complex; required for initiation of pre-meiotic DNA replication, likely due to its requirement for expression of IME1 |
| HPR1 | 4.08 | Subunit of THO/TREX complexes; this complex couple transcription elongation with mitotic recombination and with mRNA metabolism and export, subunit of an RNA Pol II complex; regulates lifespan; involved in telomere maintenance; similar to Top1p |
| DID4  (down tag) | 3.83 | Class E Vps protein of the ESCRT-III complex; required for sorting of integral membrane proteins into lumenal vesicles of multivesicular bodies, and for delivery of newly synthesized vacuolar enzymes to the vacuole, involved in endocytosis |
| BRO1 | 3.73 | Cytoplasmic class E vacuolar protein sorting (VPS) factor; coordinates deubiquitination in the multivesicular body (MVB) pathway by recruiting Doa4p to endosomes |
|  | | |

Table S2. continued

| Gene | FD^a^ | Saccharomyces Genome Database description |
| --- | --- | --- |
| MRPS16 | 3.69 | Mitochondrial ribosomal protein of the small subunit |
| VPS4 | 3.67 | AAA-ATPase involved in multivesicular body (MVB) protein sorting; ATP-bound Vps4p localizes to endosomes and catalyzes ESCRT-III disassembly and membrane release; ATPase activity is activated by Vta1p; regulates cellular sterol metabolism |
| YMR315W-A | 3.66 | Putative protein of unknown function |
| YHR022C-A | 3.61 | Putative protein of unknown function; identified by gene-trapping, microarray-based expression analysis, and genome-wide homology searching |
| GIN4 | 3.61 | Protein kinase involved in bud growth and assembly of the septin ring |
| SIP3 | 3.59 | Vacuolar H+ ATPase subunit e of the V-ATPase V0 subcomplex; essential for vacuolar acidification; interacts with the V-ATPase assembly factor Vma21p in the ER; involved in V0 biogenesis |
| FKS1 | 3.52 | Catalytic subunit of 1,3-beta-D-glucan synthase; functionally redundant with alternate catalytic subunit Gsc2p; binds to regulatory subunit Rho1p; involved in cell wall synthesis and maintenance; localizes to sites of cell wall remodeling; FKS1 has a paralog, GSC2, that arose from the whole genome duplication |
| COT1 | 3.50 | Vacuolar transporter that mediates zinc transport into the vacuole; overexpression confers resistance to cobalt and rhodium; protein abundance increases in response to DNA replication stress; COT1 has a paralog, ZRC1, that arose from the whole genome duplication |
| MIG3 | 3.50 | Transcriptional regulator; partially nonfunctional in S288C strains but has a major role in catabolite repression and ethanol response in some other strains; involved in response to toxic agents; phosphorylation by Snf1p or the Mec1p pathway inactivates Mig3p, allowing induction of damage response genesenvironment |
| VMA9 | 3.49 | Vacuolar H+ ATPase subunit e of the V-ATPase V0 subcomplex; essential for vacuolar acidification; interacts with the V-ATPase assembly factor Vma21p in the ER; involved in V0 biogenesis |
| VPS52 | 3.49 | Component of the GARP (Golgi-associated retrograde protein) complex; GARP is required for the recycling of proteins from endosomes to the late Golgi, and for mitosis after DNA damage induced checkpoint arrest; involved in localization of actin and chitin; members of the GARP complex are Vps51p-Vps52p-Vps53p-Vps54p |
| ISM1 | 3.46 | Mitochondrial isoleucyl-tRNA synthetase; null mutant is deficient in respiratory growth; human homolog IARS2 implicated in mitochondrial diseases, can partially complement yeast null mutant |
| BUD22 | 3.44 | Protein required for rRNA maturation and ribosomal subunit biogenesis; required for 18S rRNA maturation; also, required for small ribosomal subunit biogenesis; cosediments with pre-ribosomal particles; mutation decreases efficiency of +1 Ty1 frameshifting and transposition, and affects budding pattern |
| TRP3 | 3.43 | Indole-3-glycerol-phosphate synthase; forms bifunctional hetero-oligomeric anthranilate synthase: indole-3-glycerol phosphate synthase enzyme complex with Trp2p |
| EUG1 | 3.43 | Protein disulfide isomerase of the endoplasmic reticulum lumen; EUG1 has a paralog, PDI1, that arose from the whole genome duplication; function overlaps with that of Pdi1p; may interact with nascent polypeptides in the ER |
| VMS1 | 3.42 | Component of a Cdc48p-complex involved in protein quality control; exhibits cytosolic and ER-membrane localization, with Cdc48p, during normal growth, and contributes to ER-associated degradation (ERAD) of specific substrates at a step after their ubiquitination; forms a mitochondrially-associated complex with Cdc48p and Npl4p under oxidative stress that is required for ubiquitin-mediated mitochondria-associated protein degradation (MAD); conserved in C. elegans and humans |
| RER1 | 3.33 | Protein involved in retention of membrane proteins; including Sec12p, in the ER; localized to Golgi; functions as a retrieval receptor in returning membrane proteins to the ER |
| CSG2 | 3.31 | Endoplasmic reticulum membrane protein; required for mannosylation of inositolphosphorylceramide and for growth at high calcium concentrations; protein abundance increases in response to DNA replication stress |
| ^a^ Fitness defect (Log_2_ ratio) | | |


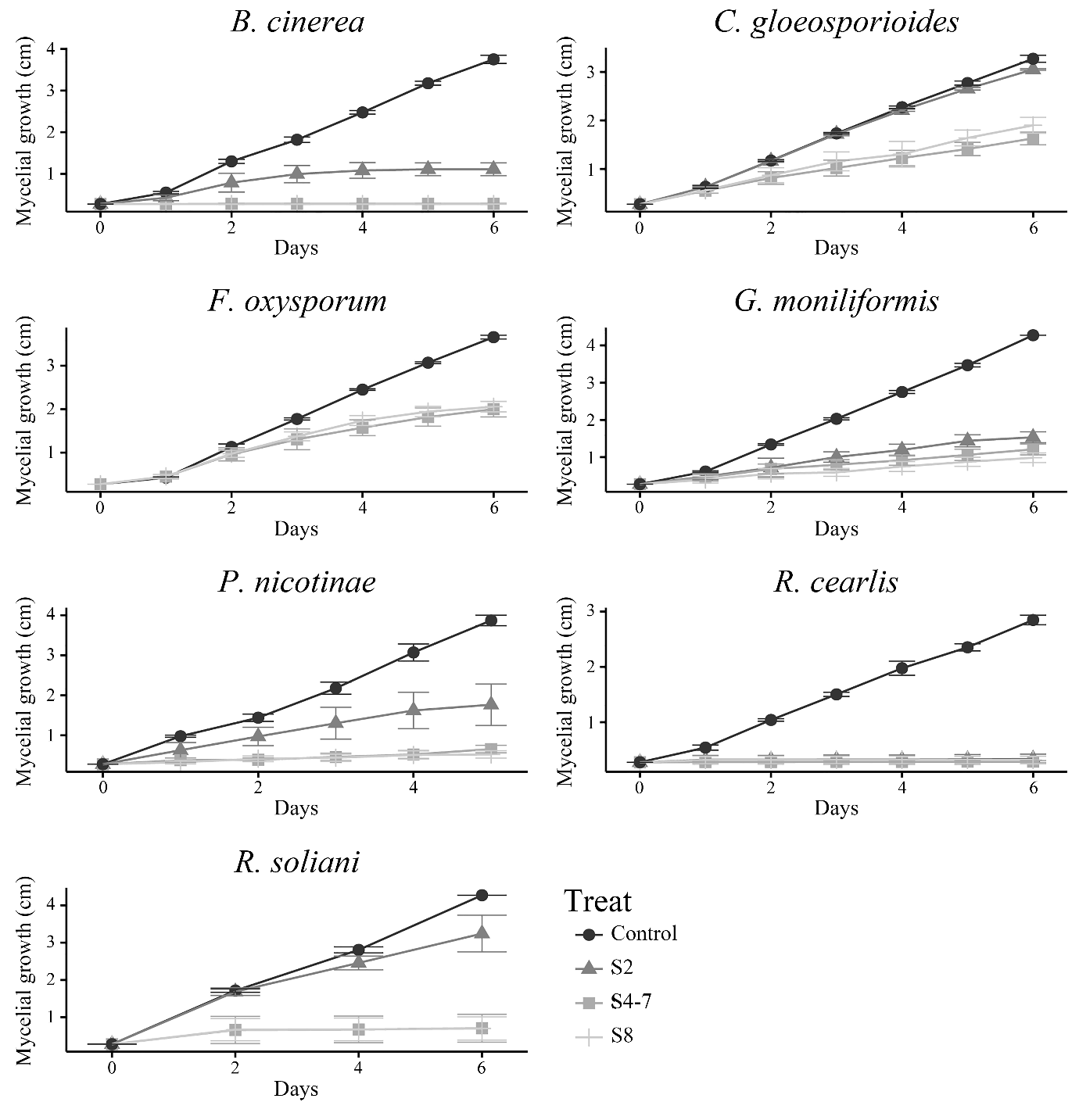


Figure S1. Mycelia growth within *Streptomyces* volatiles. S4-7 and S8 volatiles inhibit all growth. Fungi are incubated on PDK at 27 ºC and mycelia growth is measured every day. Inhibition is either less effective than others (*B*. *cinerea*, *P. nicotinae and R*. *solani*) or absent (*C. gloeosporioides*). Error bars represent standard deviations.


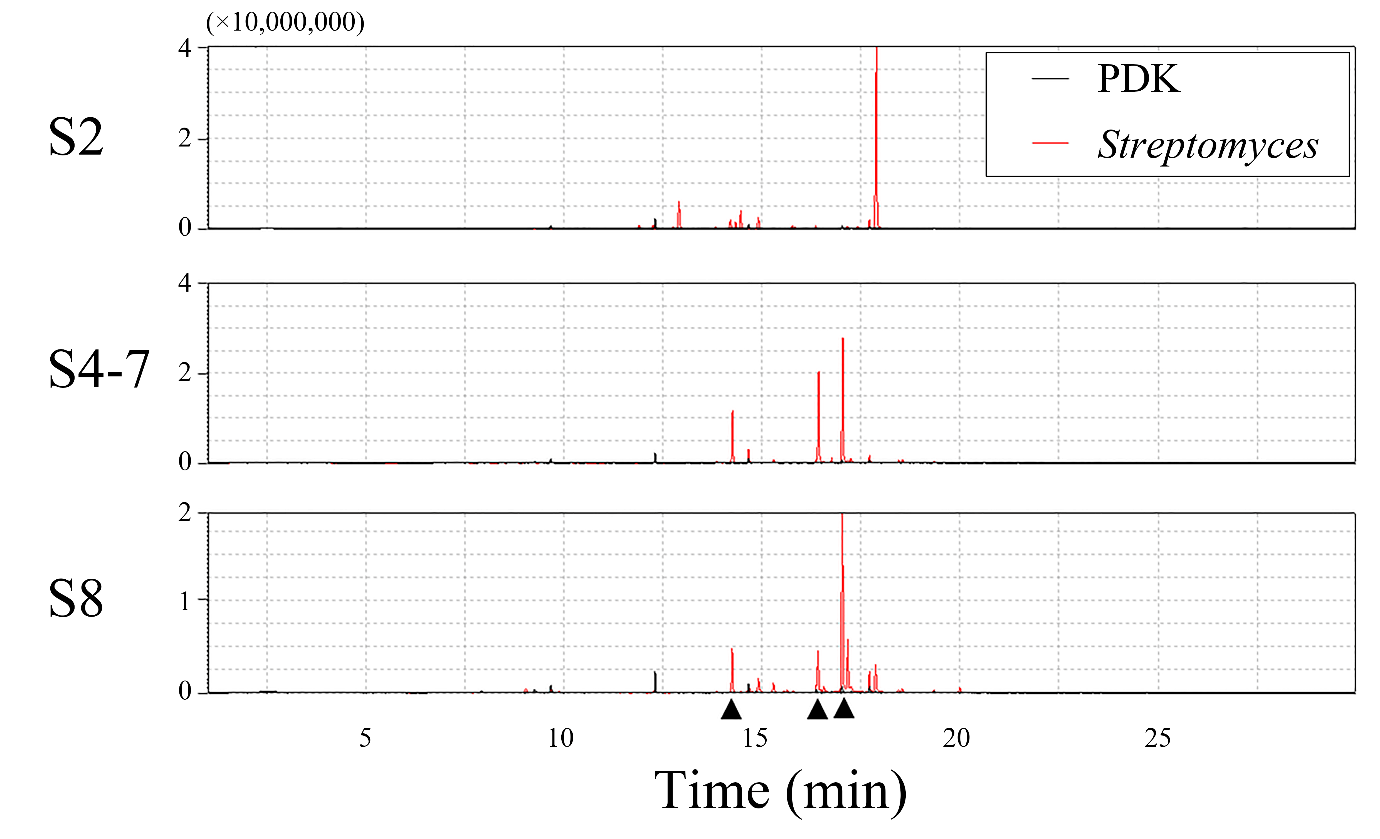


Figure S2. Comparison of GC ((EI-GC-MS; GC2010 plus-GCMS-TQ8030) total ion current. S4-7 and S8 volatiles have common peak at 14.2, 16.2 and 17.0 min.


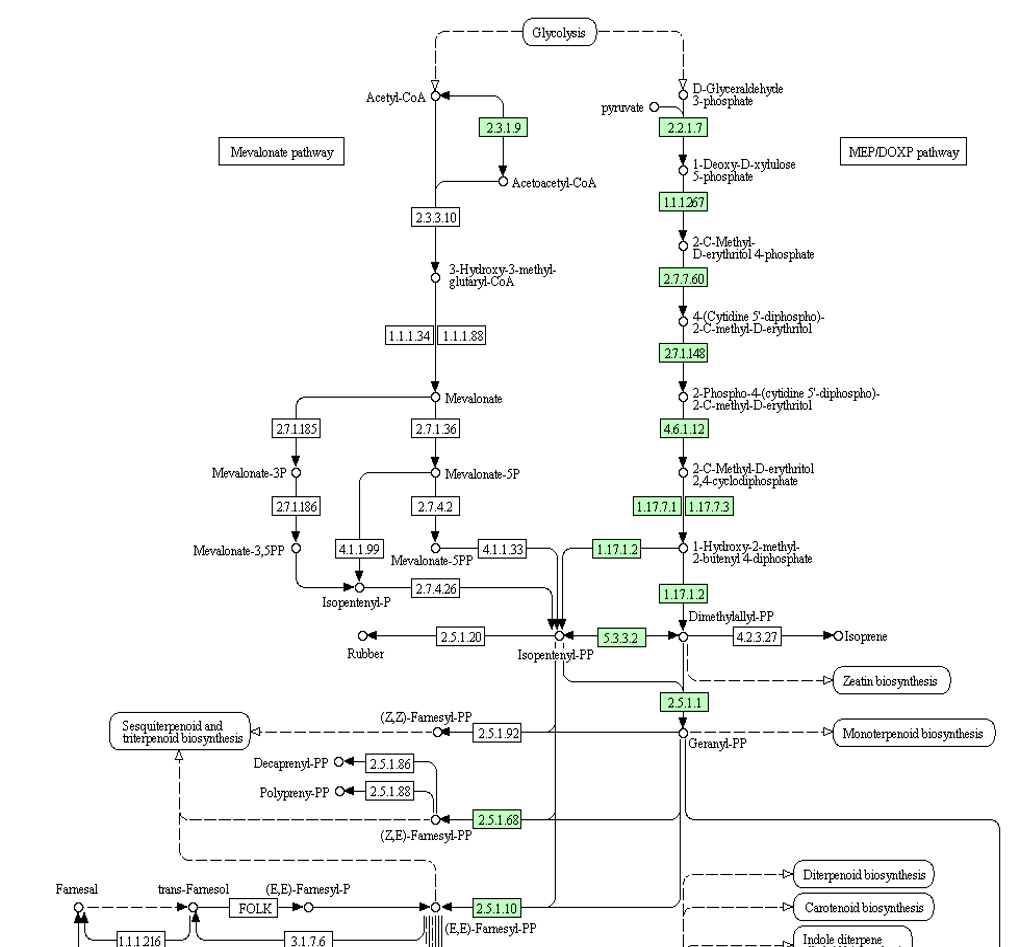


Figure S3. Sesquiterpene backbone synthesis pathway of S4-7. Gray indicates that it is in the S4-7 genome. S4-7 has sesquiterpene synthetic pathway through MEP/DOXY pathway from glycolysis.


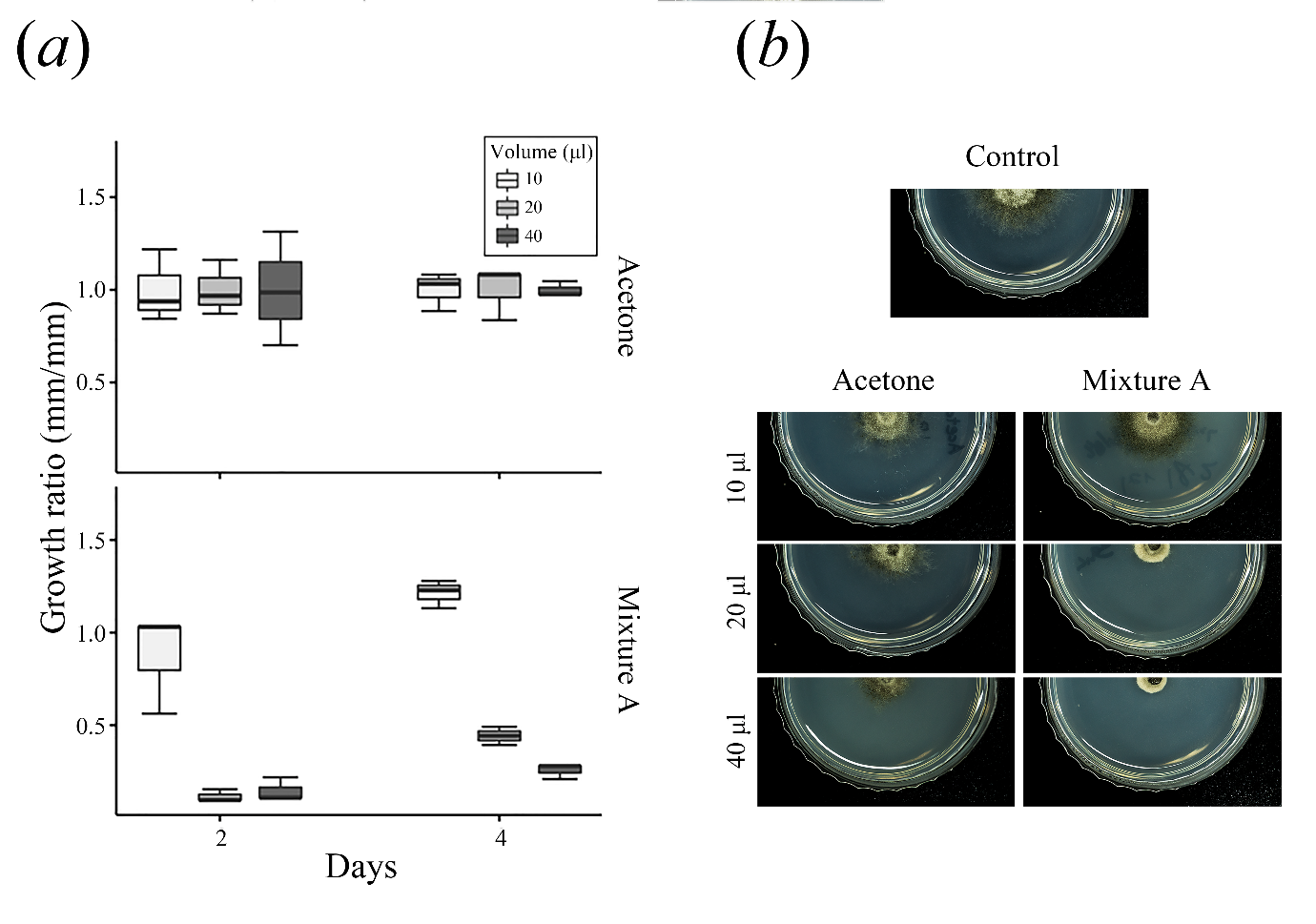


Figure S4. *B. cinerea* growth treated mixture A at its headspace. A, box-and-whisker plot of the growth ratio. B, the *B. cinerea* grows in acetone and mixture A (4 days). Mixture A inhibits growth and spore formation.


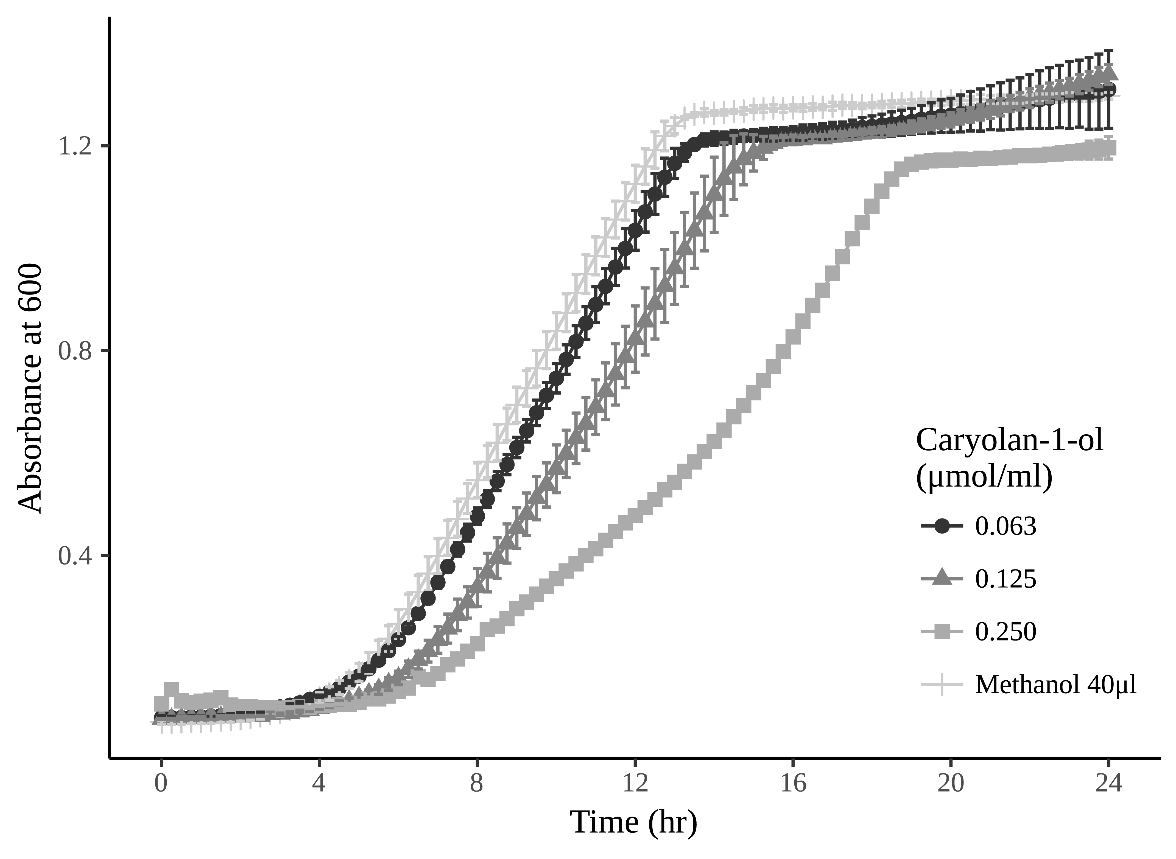


Figure S5. *S. cerevisiae* BY4743 growth curve in caryolan-1-ol. Based on control (methanol 40 μl), treatment with 0.063, 0.125 and 0.250 caryolan-1-ol μl/ml show 95.72, 88.20 and 69.20% growth, respectively.


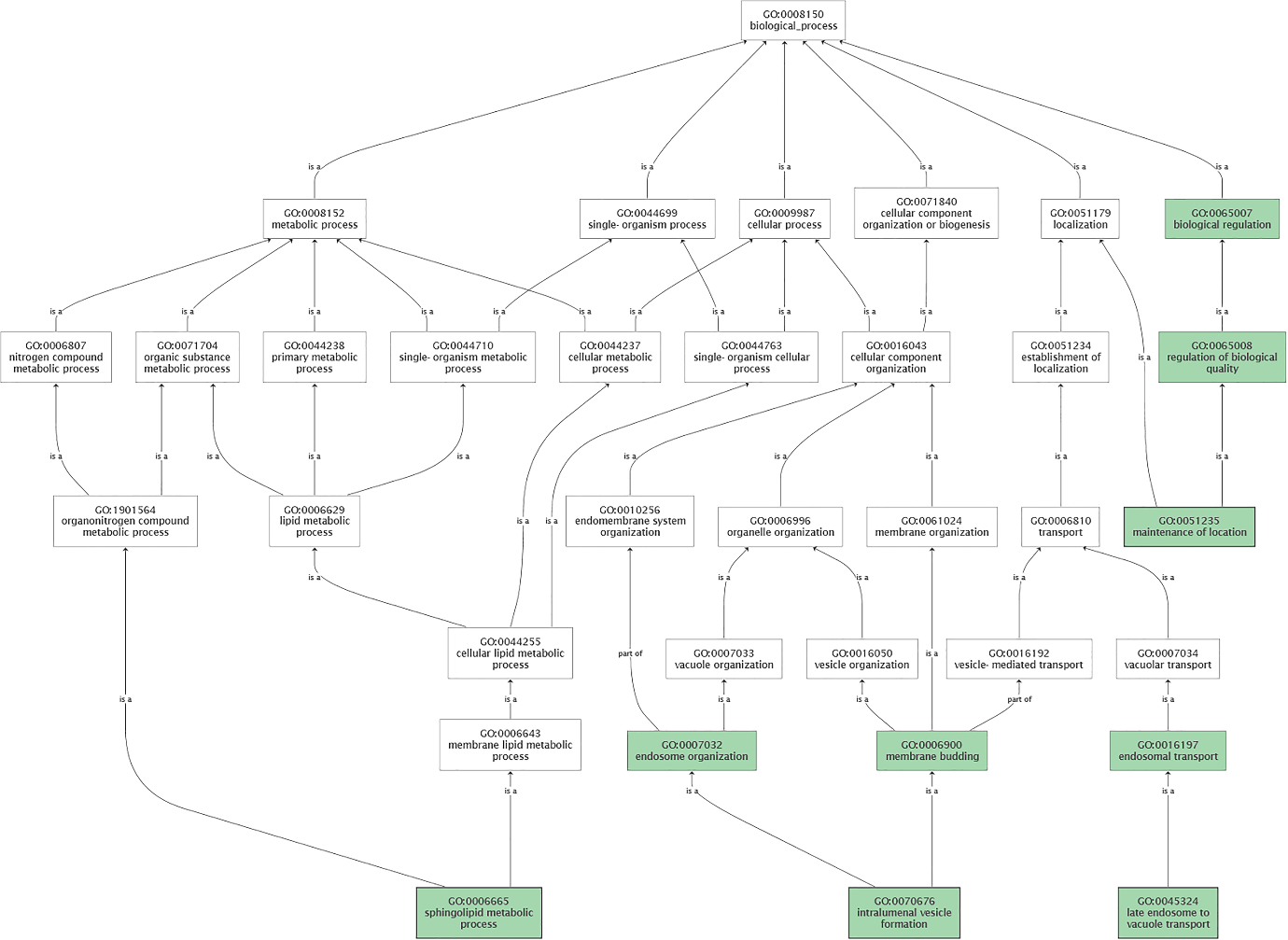


Figure S6. GO process term diagram of high defect score genes. It is drawn by Blast2GO (version 4.0.7). The terms are grouped into sphingolipid, vesicle formation, endosomal transport and maintenance of location.
